# Negative affect variability differs between anxiety and depression on social media

**DOI:** 10.1371/journal.pone.0272107

**Published:** 2024-02-21

**Authors:** Lauren A. Rutter, Marijn ten Thij, Lorenzo Lorenzo-Luaces, Danny Valdez, Johan Bollen

**Affiliations:** 1 Center for Social and Biomedical Complexity, Indiana University Bloomington, Bloomington, IN, United States of America; 2 Department of Psychological and Brain Sciences, Indiana University Bloomington, Bloomington, IN, United States of America; 3 Department of Advanced Computing Sciences, Maastricht University, Maastricht, NL, United States of America; 4 Department of Applied Health Science, School of Public Health, Indiana University Bloomington, Bloomington, IN, United States of America; 5 Luddy School of Informatics, Computing, and Engineering, Indiana University Bloomington, Bloomington, IN, United States of America; Universidad Diego Portales, CHILE

## Abstract

**Objective:**

Negative affect variability is associated with increased symptoms of internalizing psychopathology (i.e., depression, anxiety). The Contrast Avoidance Model (CAM) suggests that individuals with anxiety avoid negative emotional shifts by maintaining pathological worry. Recent evidence also suggests that the CAM can be applied to major depression and social phobia, both characterized by negative affect changes. Here, we compare negative affect variability between individuals with a variety of anxiety and depression diagnoses by measuring the levels and degree of change in the sentiment of their online communications.

**Method:**

Participants were 1,853 individuals on Twitter who reported that they had been clinically diagnosed with an anxiety disorder (*A* cohort, *n* = 896) or a depressive disorder (*D* cohort, *n* = 957). Mean negative affect (NA) and negative affect variability were calculated using the Valence Aware Dictionary for Sentiment Reasoning (VADER), an accurate sentiment analysis tool that scores text in terms of its negative affect content.

**Results:**

Findings showed differences in negative affect variability between the *D* and *A* cohort, with higher levels of NA variability in the *D* cohort than the *A* cohort, *U* = 367210, *p* < .001, *r* = 0.14, *d* = 0.25. Furthermore, we found that *A* and *D* cohorts had different average NA, with the *D* cohort showing higher NA overall, *U* = 377368, *p* < .001, *r* = 0.12, *d* = 0.21.

**Limitations:**

Our sample is limited to individuals who disclosed their diagnoses online, which may involve bias due to self-selection and stigma. Our sentiment analysis of online text may not completely capture all nuances of individual affect.

**Conclusions:**

Individuals with depression diagnoses showed a higher degree of negative affect variability compared to individuals with anxiety disorders. Our findings support the idea that negative affect variability can be measured using computational approaches on large-scale social media data and that social media data can be used to study naturally occurring mental health effects at scale.

## Introduction

Internalizing disorders, such as anxiety and depressive disorders, are the most commonly diagnosed mental illnesses [1]. They are highly comorbid due to shared vulnerabilities including genetic, temperamental, and psychological factors [2–5]. Despite their high prevalence rates and financial burden to society, many aspects of their phenomenology remain poorly understood.

In general, internalizing disorders such as generalized anxiety disorder (GAD) and major depressive disorder (MDD) are characterized by two common traits: 1) a high level of negative affect (NA) and 2) heightened NA in response to life stress [6–8]. Individuals with a variety of anxiety and mood disorders including social anxiety, for example, [9, 10], and even specific phobias [11] may experience high levels of NA at times during the course of their illness, but this heightened NA and NA reactivity are particularly associated with neuroticism [12, 13] or neurotic temperament. While the link between neuroticism and symptoms of internalizing disorders is well-established (e.g., [14]), less is known about how fluctuations in NA impact symptoms in transdiagnostic samples, and in the real world.

Indeed, in addition to decades of exploration on the structure of affect and the relation of affect to symptoms of internalizing disorders, e.g., [11, 15–18] researchers have more recently begun to examine affect variability as a concept related to symptom presentation and symptom severity in internalizing disorders and emotional well-being [19]. Affect variability, i.e. the degree to which the affect of individuals with internalizing disorders changes over time, has been linked to anxiety and depressive disorders in clinic and online samples [19–22]. Just as heart rate variability is a robust marker of physical health [23, 24], with lower variability indicating relatively better cardiovascular health, negative affect variability relates to a variety of mental health outcomes including much of the internalizing disorder spectrum [25–28]. Furthermore, NA variability is also associated with many negative health outcomes including worse sleep [29] and lowered immune response [30].

Some researchers have proposed theories as to why NA variability serves as a marker of mental health. The Contrast Avoidance Model (CAM) [31] is a relatively novel theory to explain the maintenance of pathological worry in GAD, but is now supported by substantial evidence. In fact, the CAM has recently been supported in social anxiety and major depression, as well as GAD [32]. The CAM is grounded in the idea that individuals experience the effects of an emotional experience relative to their previous emotional state (i.e., contrasts) and that negative emotional contrasts are deemed specifically unpleasant. Consequently, individuals with GAD continuously worry to maintain a stable negative emotional state, thereby reducing the probability of an unpleasant shift to a more negative emotional state (contrast avoidance) [31, 33, 34]. In sum, the CAM is based on the following ideas: 1. worry heightens negative emotion (it does not dampen activation or distress), 2. worry increases and sustains increased activation, 3. the sustained increased in negative emotion associated with worry allows for avoidance of a sharp increase in negative emotion) [31].

The CAM pertains to negative affect variability in GAD based on the idea that the maintenance of pathological worry restricts negative affect variability. This implies that negative affect variability would be higher in other internalizing disorders, such as depression, for which pathological worry is not a defining feature, even though these commonly occurring internalizing disorders share similar features with GAD, including high levels of negative affect.

This implication is noteworthy because the core processes in GAD are fundamental processes across anxiety disorders [2, 35, 36]. Moreover, GAD is highly comorbid with major depressive disorder [37], and individuals with depression commonly report excessive worry and high NA [38]. For many, a period of anxiety and worry precedes their depression [39]. Worry and rumination have been shown to mediate the relationship between neuroticism and depression and anxiety [40]. Thus, comparing negative affect variability from a framework guided by the CAM could yield novel information on the structure of affect and its variability across comorbid internalizing disorders.

To address this gap in the literature, the current study examines differences in levels and variability of expressed negative affect in a sample of online individuals who reported diagnoses of internalizing disorders. First, with respect to the levels of expressed affect, we examined differences in average NA levels between anxiety- and depression-related disorders. Second, we explore whether negative affect variability differs between anxiety and depressive disorders. Thus, our hypotheses for the current study investigated two distinct exploratory questions: (1) are there differences in mean levels of NA between depressed and anxious cohorts (based on the early finding that levels of negative emotionality are higher in individuals with depression vs. individuals with anxiety [41]), and (2) does negative affect variability differ between anxiety and depressive disorders based on an implication of the CAM [31], i.e. due to avoidance of affective shifts individuals with anxiety will show lower observed NA variability than individuals with other internalizing disorders such as depression?

To test our hypotheses, we compared NA variability (defined as the standard deviation of negative sentiment scores over time) and mean NA levels for a large sample of Twitter users who reported their clinical diagnoses of either depression or anxiety disorders. We estimate mean NA affect levels and NA variability from changes in the sentiment ratings of the tweets written by the individuals in our sample, following existing research [42, 43] that has operationalized affect variability as the standard deviation of affect over time. Computational approaches using online, longitudinal data sources (e.g., social media) may shed light onto the mechanisms and trajectories that differentiate these disorders, and identify potential targets for their treatment including emotion regulation or mindfulness based approaches, as well as text-based interventions [44–47].

## Methods

### Data gathering

We constructed our cohorts with the Indiana University Network Science Institute (IUNI) Observatory on Social Media (OSoMe, [48], a service which provides searchable access to the Twitter “Gardenhose” (a 10% sample of all daily tweets). We searched OSoMe for tweets, posted between Jan 1^st^ 2018 and Jan 1^st^ 2019, that matched both the query “diagnos*” and a specific internalizing disorder query (e.g., ‘depressed’, ‘anxiety’, ‘GAD’), which are described in Table 1. Using a previously established sample inclusion approach [44, 49], we identified a cohort of social media users who (1) received a clinical diagnosis of depression/anxiety and (2) posted an explicit report of this clinical diagnosis on Twitter, that is, by stating a variant of “I was diagnosed with depression by my doctor.” Note that these statements pertain to an actual clinical diagnosis, not a self-diagnosis. The resulting set of tweets were then filtered for matching the expressions ‘i’, ‘diagnos*’, ‘depres*/ ‘anx*’’ in that order in a case-insensitive manner, allowing insertions to match the greatest variety of diagnosis statements. We combined these queries to match the greatest variety of self-referential diagnosis statements in the obtained tweets, e.g. a tweet that states “I was in fact just ***diagnos***ed with major ***depress***ion” would match.

**Table 1:** Overview of the queries used to obtain mentions of specific internalizing disorders from OSoMe [48] and the number of individuals found using these queries. The disorders are grouped in two cohorts, depression-related disorders and anxiety-related disorders. For example, *“I’m feeling depressive* does NOT match the ‘Depression’-query as it only mentions **depressive** and not **disorder**. The number of individuals do not add up to the values in the Total row as some individuals have expressed multiple diagnoses.

Disorder	Query	Individuals
Depression	depressed, or depression, or depressive disorder	1,474
Dysthymia	dysthymia	15
Seasonal Affective Disorder	seasonal affective disorder	8
Persistent Depressive Disorder	PDD	2
Agoraphobia	agoraphobia	16
Anxiety	anxiety	1,098
Generalized Anxiety Disorder	GAD, or anxiety disorder	168
Obsessive Compulsive Disorder	OCD, or compulsive disorder	237
Panic Disorder	panic	69
Phobia	phobia	11
Total		2,389

To ensure we are only including true self-referential statements of a diagnosis of one of the internalizing disorders of interest, each tweet was assessed by three judges to exclude quotes, jokes, and external references (e.g., “My friend and I were basically diagnosed with depression after the 2016 election”). Only tweets that were unanimously rated as a self-referential statement of a clinical diagnosis by all three judges were retained for further analysis. A similar approach was deemed most accurate in a comparative analysis of social media sampling methods [50]. As is recommended [51], here, we avoid the use of data-driven supervised machine learning approaches to draw conclusions with respect to depression’s language features and its population morbidity [50].

We retrieved the timeline for each Twitter user who made a qualifying statement with respect to a diagnosis using the Twitter ‘user_timeline’ API endpoint (https://developer.twitter.com/en/docs/tweets/timelines/api-reference/get-statuses-user_timeline). Subsequently, we excluded all non-English tweets (leveraging the Twitter API machine-detected “lang” field), all retweets, and all tweets that match the diagnosis and disorder query. Finally, we also remove all tweets posted before Jan 1^st^ 2018 as these tweets predate the diagnosis reference that we obtain.

Since we were only interested in accounts belonging to individuals, we excluded accounts that M3, a demographic classification algorithm, predicted to be an organization or institution, along with other accounts that Botometer, a software that identifies automated accounts, indicated to be bots. Our data cleaning led to a final sample of *n* = 1, 853 individuals and *N* = 2, 492, 480 tweets that were posted between January 1^st^ 2018 and July 8^th^ 2020. The final column of Table 1 provides the number of individuals that were retrieved as a result of each query. Note that individuals can express multiple disorders, hence the total number of individuals is lower than the sum of individuals across all queries.

As with our prior work (e.g., [44, 45, 52]), we note that all data collection and analyses undertaken in this study complied with the user guidelines for ethical data use. Additionally, because our study involved no human interaction, and involved secondary data analysis, this study was exempt from review by the Institutional Review Board (IRB).

### Cohort construction

We grouped the individuals in our sample into two cohorts: a cohort of individuals that were diagnosed with anxiety disorders (*A* cohort) and a cohort of individuals with depressive disorders (*D* cohort). There were 536 individuals who reported diagnoses that fell into both anxiety and depressive groups, sometimes within the same tweet, sometimes in separate tweets. We did not include these individuals in the current analysis, given difficulty in identifying the relative onset of each clinical diagnosis as well as difficulty interpreting the relative effects of overall clinical severity vs. comorbidity.

The *A* cohort includes individuals with reported clinical diagnoses of agoraphobia, anxiety, GAD, obsessive compulsive disorder (OCD), panic disorder, and phobia or any combination of these disorders. Of note, we included OCD in the anxiety group because of its similarity to other anxiety disorders. The *A* cohort contained 896 individuals.

The *D* cohort contains individuals who reported diagnoses of MDD, persistent depressive disorder (PDD), seasonal affective disorder (SAD), and dysthymia (*DSM-5’s* diagnosis of persistent depressive disorder [53]) or any combination of these disorders. 957 individuals were included in this cohort.

### Demographic information

Unless an individual has specified personal demographics in their profile description or through individual tweets, Twitter accounts do not contain detailed demographic information such as age and gender. However, demographic information can be inferred from a variety of account characteristics using machine learning methods and classifying algorithms, such as M3 [54], which we used to infer demographics of Twitter accounts, and Botometer [55], which we used to remove automated accounts. The M3 system is a deep learning classifier that classifies an account along three categories; (1) gender (male/female, Macro-F1: 0.915), (2) age (“18 and below”, “19–29”, “30–39”, and “40 and up”, Macro-F1: 0.425), and (3) organization (“individual” vs “organizational account”, Macro-F1: 0.898), based on the profile image, screen name, name, and biography of an account [56]. Botometer classifies whether accounts are likely bots or not (AUC: 0.99) [57].

The M3 system and Botometer assign probabilities to each possible label to indicate their confidence that the label in question can be assigned to the individual’s account. We apply high thresholds to these probabilities to decide whether a label applies to an account or not. As a first step, we combine an account’s M3 organization and its Botometer score to remove organizations and bots from our sample. We retain all twitter accounts with a Botometer score that is lower than 0.5 (not likely a bot) and whose non-organisation score surpasses the 0.8-threshold (not likely an organization). Subsequently, we assign gender and age classifications based on the M3 outcomes for those categories (i.e., gender probability > 0.8 and age probability > 0.6) [54]. We set the threshold for age lower as this category contains four options whereas all other categories consist of two options. For more demographic information, please see Table 2.

**Table 2:** Overview of the demographic distribution of depressed (*D*) and anxious (*A*) cohorts. Note that the number of individuals in the Age or Gender categories do not sum up to the total number of individuals in the sample as some individuals do not get a clear categorization.

	Gender			Age			
	individual	male	female	18 and below	19–29	30–39	40 and up
*A* cohort	896	215	543	205	249	97	55
*D* cohort	957	277	504	225	232	91	72
Total	1,853	492	1,047	430	481	188	127

### Sentiment analysis as an indicator of affect

Following [58], we use the sentiment ratings of the tweets written by individuals (in the aggregate) to provide an indication of their negative affect. We furthermore calculate the standard deviation of the negative sentiment scores of an individual’s tweets to approximate their individual NA variability.

Text sentiment was determined using VADER [59], a widely applied sentiment analysis tool that was shown to have best-in-class accuracy in a large survey of sentiment analysis tools [60]. VADER is specifically designed to assess the valence of social media content, recognizing a wide range of common abbreviations, slang, punctuation, and other lexicographical characteristics of social media content including negation, amplification, and hedging. We chose VADER over other sentiment analysis tools for several reasons: VADER (1) was shown to have the highest accuracy in a wide-ranging benchmark of sentiment analysis tools [60], (2) was specifically designed for Twitter and social media language acknowledging a wide variety of emojis and expression that are common in the online vernacular, (3) has a large, well-vetted lexicon (each word of rated by 10 independent human evaluators), (4) is focused on general valence which covers the full spectrum, (5) also generates Negative and Positive Affect indicators, (6) explicitly recognizes a variety of important grammatical and lexical modifiers, e.g. negation (“not good”), contractions (“wasn’t very good”), hedging (“a little bit”), magnifications (“very”), punctuation (e.g. “!!!”), and acronyms (“lmao”), (7) provides auditable ratings (one can trace exactly on which grounds a rating was generated), (8) is open-source, freely available, and validated in the literature, and importantly (9) has demonstrated that its text sentiment ratings can provide an indication of the affective state of the individuals who wrote the text [58].

Since we are measuring negative affect variability in the context extending the CAM to an online sample of anxiety and depression, here we rely on VADER’s negative sentiment rating which expresses on a scale from 0 to 1 whether the content of a tweet is either entirely negative (neg = 1) or entirely positive or neutral (neg = 0). For brevity and clarity, we refer to this approximation of affect from VADER’s negative text rating as NA, or, as VADER NA scores. For a more detailed description of VADER’s workings, we refer the reader to the S1 Appendix.

### Measuring negative affect variability

For each individual in our sample, we determined the negative VADER ratings (here referred to as VADER NA) of each tweet in the entire collection of their individual tweets. We then calculated both the *level* of NA as the average of *within-subject* VADER NA scores, and the *spread*, as the standard deviation of *within-subject* VADER NA scores, of expressed affect in our obtained cohorts.

### Bootstrapping

To account for the variability of individual behavior in our sample, we bootstrapped our analyses. The bootstrap was performed by randomly re-sampling the individuals of a given cohort, with replacement, until the size of the sample obtained is the same as that of the original cohort. Next, we calculated the desired quantity (e.g., the median activity of the sample). By repeating this procedure 10000 times, each re-sample yields an estimation of the desired quantity. The distribution of 10000 estimations of the quantity indicates the range of the desired quantity given random variations in the underlying sample (e.g., the median activity of a cohort).

### Statistical analysis

Our statistical analysis consisted of two parts. First, we investigated the difference in activity between both cohorts. As the average number of tweets per day spanned multiple orders of magnitude, we compared activity levels across the cohorts using a Mann-Whitney U non-parametric test. Second, we compared both the level (within-subject average) and spread (within-subject standard deviation) of VADER NA between the *A* and *D* cohorts with the non-parametric Mann-Whitney U test and we determined the effect size using Cohen’s *d*.

## Results

We compare our *A* and *D* cohorts in terms of (1) their online activity levels, (2) the degree to which they have similar or different overall levels of NA, and (3) the degree to which the *A* and *D* cohort have similar or different levels of NA variability.

### Online activity levels between *A* and *D* cohorts

Because differences in activity levels could influence affect variability, we first verify whether the individuals *A* and *D* cohorts have similar activity levels. For each individual we calculate their average number of tweets per day by dividing the total number of tweets in their timeline by the length of the timeline in days. This average number of tweets per day serves as an indicator of the individual’s online activity level. We performed a Mann-Whitney U test to compare the distributions of these individual activity levels per day (4.02 and 3.94 tweets per day for the *A* and *D* cohorts, respectively) between the *A* and *D* cohort, respectively, and failed to reject the null-hypothesis that the distributions are identical (*U* = 441351, *p* = .273, *r* = −0.029), meaning that we did not find statistically significant differences in online activity levels between the two cohorts and that it is unlikely to influence our analysis.

### Negative affect levels between *A* and *D* cohorts

**Fig 1:**
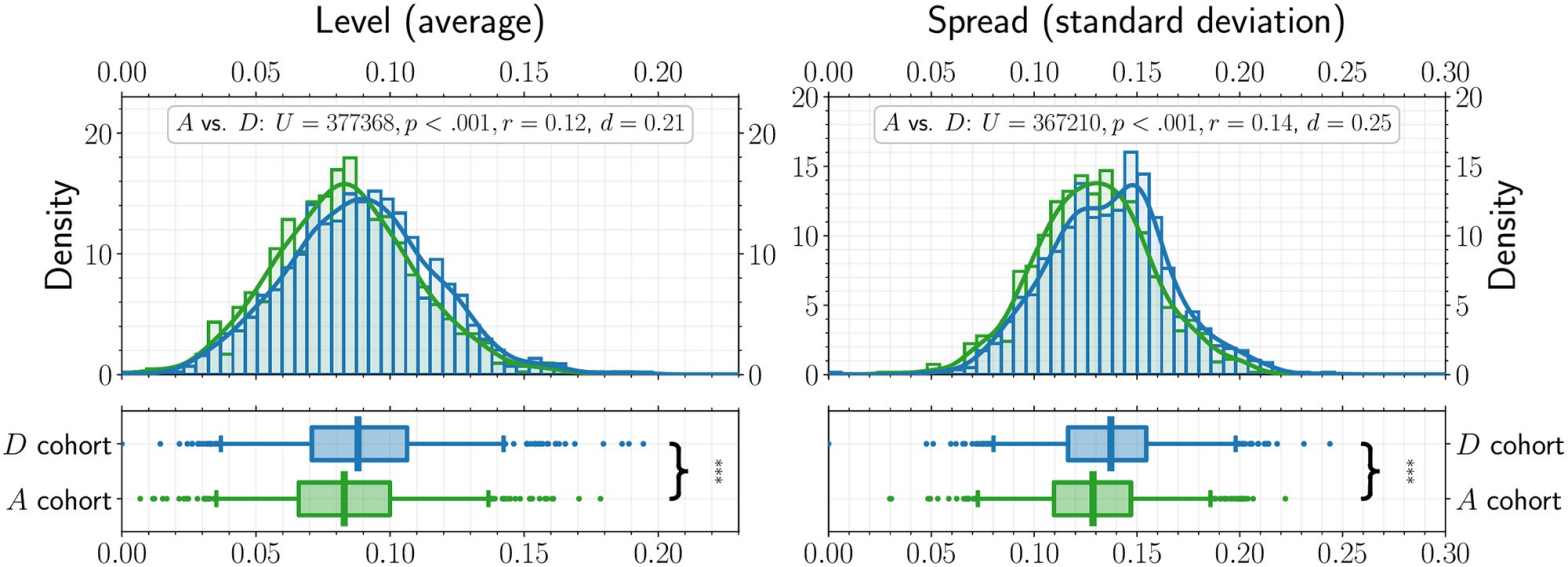
Mean negative affect and negative affect variability comparison between the *A* (anxiety-related disorders, *n* = 896) and *D* (depression-related disorders, *n* = 957) cohorts. Distributions of *within-subject level* (average) and *spread* (standard deviation) of negative affect (NA) as measured with VADER [59]. Each comparison is displayed in two panels. The top panel displays a histogram and a kernel density estimate (KDE) of the distribution (solid line). The bottom panel shows a box-and-whisker (box: 50% CI, whisker: 95% CI) plot of the distribution. All points that fall outside the 95% CI are indicated by dots. The vertical line within the box displays the median value.

We hypothesized that NA levels would differ between the *A* and *D* cohorts and that the *A* cohort would have lower NA levels than the *D* cohort. We therefore perform a comparison of the mean NA levels for the individuals in the *A* and *D* cohorts as shown in Fig 1. The result of a Mann-Whitney U test indicate that NA levels do differ between the two groups (***, *U* = 377368, *p* < .001, *r* = 0.12, *d* = 0.21) and that NA levels are indeed higher for the *D* cohort than the *A* cohort (mean NA levels of 0.083 and 0.089, respectively). In other words, our analysis suggests that individuals with depression have significantly higher levels of NA on average than individuals with anxiety disorder.

### Negative affect variability between *A* and *D* cohorts

We hypothesized that negative affect variability would differ between our *A* and *D* cohorts because individuals with anxiety, according to the CAM, may avoid affective shifts by maintaining pathological worry. We quantified the difference in negative affect variability between our *D* and *A* cohorts by calculating the standard deviation of the distribution of NA values for each of the individuals in the *A* and *D* cohort. The observed *within-subject* NA standard deviations (referred to as NA spread) averages were 0.13 for the *A* cohort and 0.14 for the *D* cohort, respectively. We performed a Mann-Whitney U test to determine whether the distributions of the individual standard deviations differed between the *A* and *D* cohorts. We found that we could reject the null-hypothesis that the distributions are identical (***, *U* = 367210, *p* < .001, *r* = 0.14, *d* = 0.25), indicating that NA variability of the *D* cohort is higher than that of the *A* cohort, and that the *A* cohort is characterized by *lower* NA variability than the *D* cohort.

### Robustness check with compound scores and positive affect

We include *A* and *D* cohort comparisons based on VADER’s positive measurements (PA score), see S1 Appendix “Analysis of VADER positive scores”, and VADER’s compound score (both negative and positive combined) in the S1 Appendix “Analysis of VADER compound scores”. We caution that these measurements are not congruent with the CAM since the latter is concerned with negative affect variability. In fact, one would expect to find no significant differences in positive affect variability between the *A* and *D* cohorts and a more minor effect for the VADER compound measurement since it combines a measurement of positive and negative affect thereby attenuating the possible effect of NA variability. As shown in the S1 Appendix, this is borne out by our results: we find (1) no significant differences in positive affect (PA) variability and (2) a smaller, marginally significant difference when comparing the compound score between the *A* and *D* cohorts.

### Variations between individuals

We also performed a bootstrap analysis to verify that our results are robust to random variations in the composition of our *A* and *D* cohorts as shown in Table 3. This bootstrap resampled the individuals in our *A* and *D* cohorts with replacement and reaffirmed our earlier reports; the confidence intervals (CIs) for NA level and spread do not overlap, indicating a significant difference in the distributions of expected NA level and NA variability.

**Table 3:** Outcomes of the bootstrap analysis comparing all considered affect measures in depressed (*D*, *n* = 957) vs. anxious (*A*, *n* = 896) cohorts. Measures used are *within-subject level* (average) and *spread* (standard deviation) of NA. All scores are calculated with VADER [59]. The ‘*A* vs. *D*’-column displays the significance of the comparison between the *A* (anxiety-related disorders) and *D* (depression-related disorders) cohorts (*ns*: *p* ≥ .05 *: *p* < .05, **: *p* < .01, and ***: *p* < .001).

Bootstrapped quantity	Cohort	Measure	CI	*A* vs. *D*
Median # tweets per day	*A*	*Mdn* = 4.02	[3.51, 4.57]	*ns*
	*D*	*Mdn* = 3.94	[3.37, 4.44]	
Mean *within-subject* level of NA	*A*	*M* = 0.083	[0.082, 0.085]	***
	*D*	*M* = 0.089	[0.087, 0.091]	
Mean *within-subject* spread of NA	*A*	*M* = 0.13	[0.13, 0.13]	***
	*D*	*M* = 0.14	[0.13, 0.14]	

## Discussion

This study examined differences in mean negative affect and negative affect variability in a large, online sample of Twitter users with anxiety and depressive disorder diagnoses. Our results provided support for both of our study hypotheses. Our first exploratory hypothesis was that the levels of NA would differ between the individuals in the anxiety (*A*) and depression (*D*) cohorts. Indeed, the level of NA is lower for the *A* cohort compared to the *D* cohort (Fig 1). Our second hypothesis was that NA variability would differ between individuals with depression and anxiety disorders. Here, we found that NA variability was lower for the *A* cohort compared to the *D* cohort (Fig 1). The CAM, which was formulated for GAD, suggests that because individuals with GAD seek to avoid negative emotional contrasts, NA levels and NA variability may differ between anxiety and depression cohorts, and our results indicate this is indeed the case. Our results support the notion that depression and anxiety are distinct disorders associated with different levels of NA variability and possible different mechanisms that involve worry and changes in affect. However, future work should explore this connection taking into account the time-varying nature of these signals, since we only analyze the affect scores on a *within-subject* level. In addition, different types of sentiment ratings could be explored. We show a similar analysis in the S1 Appendix using VADER’s positive and compound ratings. Since anxiety and depression are marked by negative affect, we did not expect and do not find a difference of positive affect levels and PA variability in this case. Moreover, we find a smaller, marginally significant effect for the compound score, supporting the notion that the CAM pertains to negative affect variability. However, future research may involve a variety of affect indicators to elucidate the variegated role of affect in internalizing disorders.

Beyond these primary findings, our study draws needed insight into alternative data sources to study mental disorders, as many other researchers have done (e.g., [49, 51, 61]). Indeed, we successfully leveraged computational methods and large-scale social media data to investigate the dynamics of internalizing disorders, with no participant burden. Participants in this study filled out no questionnaires or did not participate in clinical interviews. Rather, we use a sentiment analysis tool (VADER) to capture NA and NA variability to differentiate groups of individuals with internalizing psychopathology from a computational perspective; this allowed us to aggregate longitudinal, free-response data among individuals with documented mental health disorders. Although it is currently not feasible to accurately diagnose individuals with mental disorders from social media feeds, previous studies have demonstrated the feasibility of relying on online reports of actual clinical diagnoses [44].

This research approach raises a number of complex ethical questions. Data from social media platforms such as Twitter is specifically intended to be publicly available and shared as such by its users. However, individuals do not necessarily realize that this information can be used for retrospective analysis at the time of posting, nor is it clear that they would necessarily consent to such analysis [62]. Consequently, we limit our analysis strictly to comparisons of aggregate information of the de-identified cohorts of individuals to protect user privacy.

Our results suggest that social media can be usefully leveraged to study mental health for large populations of online individuals. With large pluralities of the world’s population connected to the internet and participating in social media platforms, this raises the possibility of investigating additional questions with respect to the genesis and dynamics of mental health disorders at the individuals as well as societal level. We recently demonstrated that more negative affect and depression is associated with diverging sleep patterns [45] and greater expression of cognitive distortions [44]. We have also shown a decline in affect during the COVID-19 pandemic [46], and how declining affect may be related to social inequities [52]. This current work is an expansion on prior literature because it is a large transdiagnostic (across multiple diagnoses) sample. Additionally, the present analysis was conducted in the aggregate over three years of individual timeline data, but, since date-time stamps of tweets are provided in second-resolution (ISO-8601), our approach would in principle allow the tracking of affective changes at the resolution of days, weeks, and years, thus enabling future investigations of diachronic affects in the development and trajectories of internalizing disorders. Twitter is well-suited to conduct this type of research as it allows to measure variability over long time periods, uses the individual’s own natural language, requiring no active data input (questionnaires or behavioral tasks). However, recent changes in the availability of the Twitter data may affect our ability to conduct this line of research.

Despite the strengths of our study including our large, transdiagnostic sample, significant challenges and limitations may need to be overcome. A sample that reports their clinical diagnoses may be subject to self-selection bias of individuals willing to disclose their diagnoses. The stigma associated with these disorders may play a role as well. Importantly, we must reiterate that in our data set and analyses, individuals did not self-diagnose by their own opinion. They self-selected to the study by posting a tweet that explicitly stated they were clinically diagnosed by a mental health professional or doctor which was evaluated and adjudicated as a valid report of a clinical diagnosis by three expert raters. This approach was shown to be most accurate in a comparative analysis of social media sampling methods [50]. We note that only individuals with the ability to (1) obtain a clinical diagnosis and (2) publicly share their diagnosis online were therefore included in this study. This may exclude individuals with negative self-stigma. For example, those with certain internalizing disorders may be less likely to reveal diagnoses online. It is not clear whether or how those who self-report diagnoses differ from those who do not, with respect to the factors we investigated in this study. For some individuals, revealing a diagnosis may be met with positive support (communities online and self-help), while others may experience shame and stigmatization. Revealing a mental disorder online may be particularly challenging for some minority groups including individuals who are transgender [63]. These social and psychological biases on the basis of large-scale surveys and screenings of individuals who volunteer their Twitter handles should be further explored in future work. In our own recent work we showed that individuals are generally accurate in their ability to self-diagnose common conditions like depression and anxiety disorders [64], as those who have been diagnosed and those who believe they should be diagnosed have similar scores on self-report questionnaires. Additionally, a higher degree of precision in demographic variables is warranted, as the M3 classifier that we used is not without error. Moreover, despite the strengths of VADER, a different sentiment analysis tool may have yielded different results. Another limitation of the current work is that we did not assess all internalizing disorders (i.e., trauma and related disorders such as posttraumatic stress disorder are missing), and we did not control for psychiatric or medical comorbidities, which certainly play a role in expressed negative affect and NA variability. Future work should expand analyses such as these to include the full spectrum of internalizing and externalizing psychopathology, as well as the study of NA variability and contrast avoidance in specific disorders.

## Supporting information

S1 Appendix A detailed description of the workings of VADER and the results of an analysis of VADER positive measurements and VADER compound scores. (ZIP)
