# Supplementary material for: Negative affect variability differs between anxiety and depression on social media
Source: PLoS One. 2024 Feb 21;19(2):e0272107. doi: 10.1371/journal.pone.0272107 (PMC10881019; doi:10.1371/journal.pone.0272107)
Supplement: S1 Appendix — (ZIP) [file pone.0272107.s001.zip › S1_Appendix.pdf]

S1 Appendix: Supplementary Material

This is the Supplementary Material for the manuscript titled 'Negative affect variability differs between anxiety and depression on social media'. Here, we discuss two topics: a detailed description of the workings of VADER and the results of our analysis of VADER positive measurements and VADER compound scores.

Description of VADER sentiment ratings

For each individual message, VADER outputs a compound valence score, a value ranging from -1 (i.e., highly negative sentiment) to +1 (i.e., highly positive sentiment). VADER scores can also be 0, indicating neutrality (i.e., neutral affect/sentiment). In addition to this compound score, VADER also provides information on which ratio of the total score of the text relates to positive (pos), negative (neg), and neutral (neu) aspects of the compound score. To further explore this, we will look at several examples that are displayed in Table S1.

Table S1: **Example of VADER scoring output.**

Example sentent	VADER output			
	compound	pos	neu	neg
"Gloomy day, only despair and disappointment."	-0.7351	0.0	0.294	0.706
"The boy's a liar"	-0.5106	0.0	0.476	0.524
"You're not lookin' at me"	0.0	0.0	1.0	0.0
"Take a look inside your heart"	0.6369	0.457	0.543	0.0
"Embracing beauty, surrounded by joy and love."	0.9153	0.747	0.253	0.0
"Today is a good day. Yesterday was a bad day."	-0.1531	0.201	0.556	0.243

As shown in all examples, the pos, neu and neg ratings range from 0 to 1, and contain information about the overall valence dimension because it is calculated from the same lexicon in which each word was rated from "extremely negative" to "extremely positive" (i.e. the entire range), and calculated in a manner such that the pos, neu and neg ratings sum to one. In other words, when neg has a high value, pos or neu will be low, and vice versa. See Table S2 for a more detailed description of how these values are determined.

Analysis of VADER positive measurements

In addition to VADER negative measurements (NA score), we also perform an analysis of VADER positive measurements (PA score) both in terms of the observed *within-individual* levels and *within-individual* spread.

First, we calculated the mean of the observed *within-subject* average of VADER PA scores (referred to as PA level) to gauge differences in PA level between the two cohorts. We calculated mean levels of $Mdn = 0.16$ and $Mdn = 0.15$ for the A and D cohorts, respectively. To compare PA level (average) across groups, we performed a Mann-Whitney U test. We found higher median PA levels for the A cohort compared to the D cohort *, $U = 457934, p = .011, r = -0.068$, $d = 0.090$), although the effect size is very small.

The median values of *within-subject* standard deviations of VADER PA scores (referred to as PA spread) were 0.18 for the A cohort and 0.18 for the D cohort, respectively. To compare PA spread (standard deviation) across the groups, we performed a Mann-Whitney U test. We found no evidence of a difference in the spread of PA between the D cohort and the A cohort (ns, $U = 429236, p = .965, r = -0.001$, $d = 0.003$).

Table S2: **Example of VADER scoring mechanisms.** For this example, the text "Today is a good day. Yesterday was a bad day." is used. Since this text does not contain any booster words, we can look up all individual words in the VADER-lexicon. Both *'good'* and *'bad'* are contained in this lexicon, with their scores being 1.9 and -2.5, respectively. To account for the fact that some words have no score, VADER applies +1 smoothing when calculating the pos, neu and neg scores. The exact formulas used to calculate the pos, neu and neg scores are provided below.

	today	is	a	good	day	yesterday	was	a	bad	day	+1-smoothing
pos: s_+	-	-	-	1.9	-	-	-	-	-	-	$1.9 + 1 = 2.9$
neu: s_n	0	0	0	-	0	0	0	0	-	0	$0 + 8$
neg: s_-	-	-	-	-	-	-	-	-	2.5	-	$2.5 + 1 = 3.5$

$$PA = \frac{s_+}{s_+ + s_n + s_-} = 2.5/14.4 = 0.201$$

$$NA = \frac{s_-}{s_+ + s_n + s_-} = 3.5/14.4 = 0.243$$

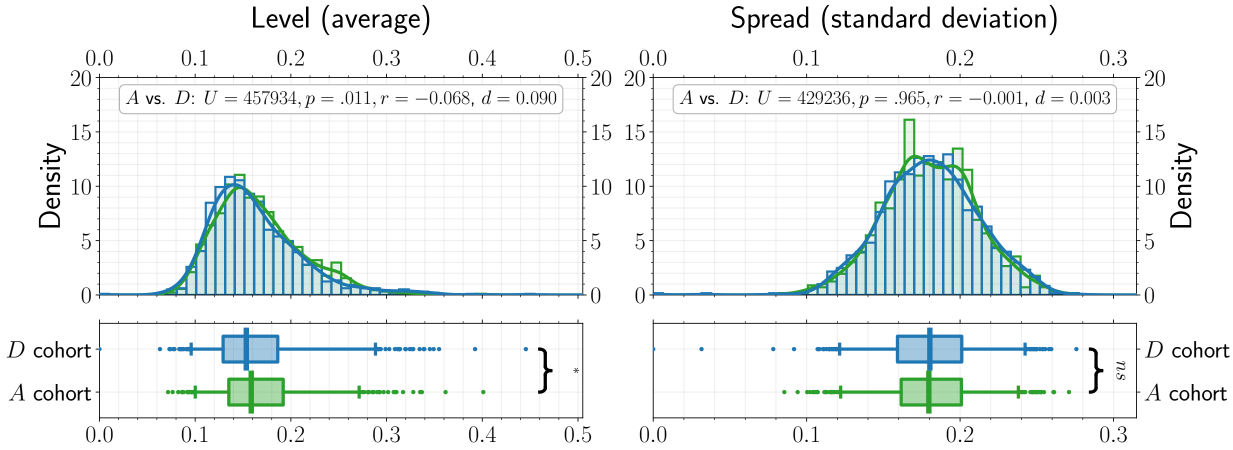

Figure S1: **Mean affect and affect variability comparison between the A (anxiety-related disorders, $n = 896$) and D (depression-related disorders, $n = 957$) cohorts.** Distributions of *within-subject level* (average) and *spread* (standard deviation) of VADER positive measurements (PA score) as measured with VADER [1]. Each comparison is displayed in two panels. The top panel displays a histogram and a kernel density estimate (KDE) of the distribution (solid line). The bottom panel shows a box-and-whisker (box: 50% CI, whisker: 95% CI) plot of the distribution. All points that fall outside the 95% CI are indicated by dots. The vertical line within the box displays the median value.

Variations between individuals

In addition to these statistical comparisons, we also performed a bootstrap analysis to account for the variability of individual behavior in our samples. The results of this bootstrap for the spread and level of affect scores are shown in Table S4. This bootstrap analysis reaffirmed our earlier results, as the confidence intervals (CIs) for PA level and spread overlap, giving no evidence for a difference between both cohorts.

Table S3: **Outcomes of the bootstrap analysis comparing all considered affect measures in depressed (D, $n = 957$) vs. anxious (A, $n = 896$) cohorts.** Measures used are *within-subject level* (average) and *spread* (standard deviation) of PA. All scores are calculated with VADER [1]. The 'A vs. D'-column displays the significance of the comparison between the A (anxiety-related disorders) and D (depression-related disorders) cohorts (*ns*: $p \geq .05$ *: $p < .05$, **: $p < .01$, and ***: $p < .001$).

Bootstrapped quantity	Cohort	Measure	CI	A vs. D
Median *within-subject* level of PA	A	$Mdn = 0.17$	$[0.16, 0.17]$	*ns*
	D	$Mdn = 0.16$	$[0.16, 0.17]$	
Median *within-subject* spread of PA	A	$Mdn = 0.18$	$[0.18, 0.18]$	*ns*
	D	$Mdn = 0.18$	$[0.18, 0.18]$	

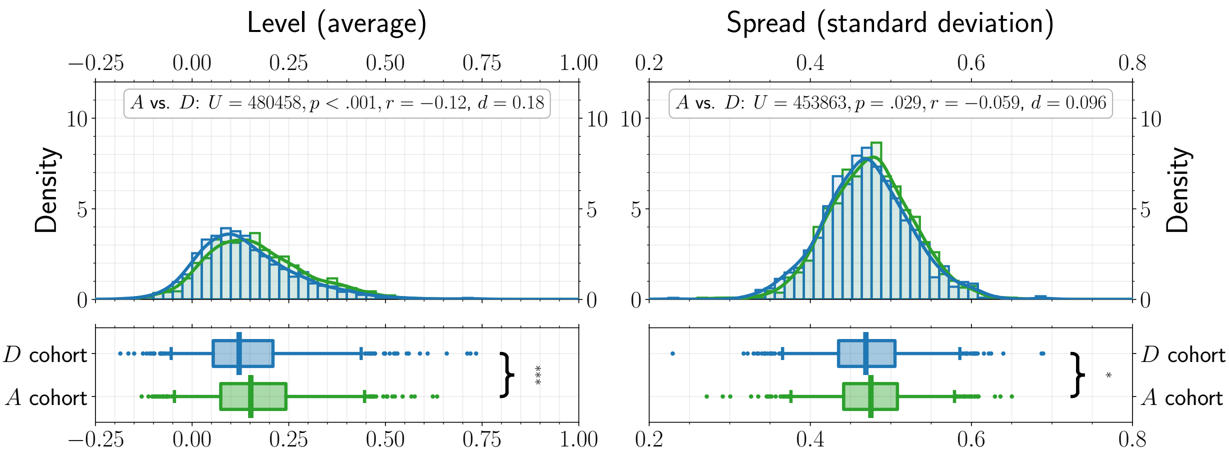

Figure S2: **Mean affect and affect variability comparison between the A (anxiety-related disorders, $n = 896$) and D (depression-related disorders, $n = 957$) cohorts.** Distributions of *within-subject level* (average) and *spread* (standard deviation) of compound scores as measured with VADER [1]. Each comparison is displayed in two panels. The top panel displays a histogram and a kernel density estimate (KDE) of the distribution (solid line). The bottom panel shows a box-and-whisker (box: 50% CI, whisker: 95% CI) plot of the distribution. All points that fall outside the 95% CI are indicated by dots. The vertical line within the box displays the median value.

Analysis of VADER compound scores

In addition to VADER negative and positive measurements, we also perform an analysis of VADER compound scores both in terms of the observed *within-individual* levels and *within-individual* spread.

First, we calculated the mean of the observed *within-subject* average of VADER compound scores (referred to as compound level) to gauge differences in compound level between the two cohorts. We calculated mean levels of $Mdn = 0.15$ and $Mdn = 0.12$ for the A and D cohorts, respectively. To compare compound level (average) across groups, we performed a Mann-Whitney U test. We found higher median compound levels for the A cohort compared to the D cohort (***, $U = 480458, p < .001, r = -0.12$, $d = 0.18$).

The median values of *within-subject* standard deviations of VADER compound scores (referred to as compound spread) $Mdn = 0.48$ and $Mdn = 0.47$ for the A and D cohorts, respectively. To compare compound spread (standard deviation) across the groups, we performed a Mann-Whitney U test. We found a smaller, marginally significant difference in the spread of compound scores between the D cohort and the A cohort (*, $U = 453863, p = .029, r = -0.059$, $d = 0.096$).

Variations between individuals

In addition to these statistical comparisons, we also performed a bootstrap analysis to account for the variability of individual behavior in our samples. The results of this bootstrap for the spread and level of affect scores are shown in Table S4. This bootstrap analysis reaffirmed our earlier results, as the confidence intervals (CIs) for PA level and spread overlap, giving no evidence for a difference between both cohorts.

Table S4: **Outcomes of the bootstrap analysis comparing all considered affect measures in depressed (D, $n = 957$) vs. anxious (A, $n = 896$) cohorts.** Measures used are *within-subject level* (average) and *spread* (standard deviation) of PA. All scores are calculated with VADER [1]. The 'A vs. D'-column displays the significance of the comparison between the A (anxiety-related disorders) and D (depression-related disorders) cohorts (ns: $p \geq .05$ *: $p < .05$, **: $p < .01$, and ***: $p < .001$).

Bootstrapped quantity	Cohort	Measure	CI	A vs. D
Median *within-subject* level of compound scores	A	$Mdn = 0.17$	$[0.16, 0.17]$	***
	D	$Mdn = 0.14$	$[0.13, 0.15]$	
Median *within-subject* spread of compound scores	A	$Mdn = 0.48$	$[0.47, 0.48]$	*
	D	$Mdn = 0.18$	$[0.47, 0.47]$	

References

[1] Clayton J Hutto and Eric Gilbert. VADER: A Parsimonious Rule-based Model for Sentiment Analysis of Social Media Text. In *Proceedings of the Eighth International AAAI Conference on Weblogs and Social Media*, ICWSM, pages 216–225. ACM, 6 2014. URL https://ojs.aaai.org/index.php/ICWSM/article/view/14550.
